# Epicardial Adipose Tissue and Cardiac Arrhythmias: Focus on Atrial Fibrillation

**DOI:** 10.3389/fcvm.2022.932262

**Published:** 2022-06-30

**Authors:** Maddalena Conte, Laura Petraglia, Serena Cabaro, Vincenza Valerio, Paolo Poggio, Emanuele Pilato, Emilio Attena, Vincenzo Russo, Adele Ferro, Pietro Formisano, Dario Leosco, Valentina Parisi

**Affiliations:** ^1^Department of Translational Medical Sciences, University of Naples Federico II, Naples, Italy; ^2^Casa di Cura San Michele, Maddaloni, Italy; ^3^Centro Cardiologico Monzino IRCCS, Milan, Italy; ^4^Department of Advanced Biomedical Science, University of Naples Federico II, Naples, Italy; ^5^Department of Cardiology, Monaldi Hospital, Naples, Italy; ^6^Chair of Cardiology, Department of Translational Medical Sciences, University of Campania “Luigi Vanvitelli” – Monaldi and Cotugno Hospital, Naples, Italy; ^7^Institute of Biostructure and Bioimaging, Consiglio Nazionale delle Ricerche, Naples, Italy

**Keywords:** epicardial adipose tissue, visceral fat, inflammation, atrial fibrillation, fibrosis, atrial remodeling, arrhythmogenesis

## Abstract

Atrial Fibrillation (AF) is the most frequent cardiac arrhythmia and its prevalence increases with age. AF is strongly associated with an increased risk of stroke, heart failure and cardiovascular mortality. Among the risk factors associated with AF onset and severity, obesity and inflammation play a prominent role. Numerous recent evidence suggested a role of epicardial adipose tissue (EAT), the visceral fat depot of the heart, in the development of AF. Several potential arrhythmogenic mechanisms have been attributed to EAT, including myocardial inflammation, fibrosis, oxidative stress, and fat infiltration. EAT is a local source of inflammatory mediators which potentially contribute to atrial collagen deposition and fibrosis, the anatomical substrate for AF. Moreover, the close proximity between EAT and myocardium allows the EAT to penetrate and generate atrial myocardium fat infiltrates that can alter atrial electrophysiological properties. These observations support the hypothesis of a strong implication of EAT in structural and electrical atrial remodeling, which underlies AF onset and burden. The measure of EAT, through different imaging methods, such as echocardiography, computed tomography and cardiac magnetic resonance, has been proposed as a useful prognostic tool to predict the presence, severity and recurrence of AF. Furthermore, EAT is increasingly emerging as a promising potential therapeutic target. This review aims to summarize the recent evidence exploring the potential role of EAT in the pathogenesis of AF, the main mechanisms by which EAT can promote structural and electrical atrial remodeling and the potential therapeutic strategies targeting the cardiac visceral fat.

## Introduction

As global average life expectancy increased, there has been an increase in the incidence and prevalence of atrial fibrillation (AF). AF affects 2.5% of the world’s population, with a prevalence reaching 10% in people over the age of 80 (1, 2). AF is a progressive and multifactorial arrhythmia, frequently intertwined with the most common cardiovascular diseases, with which it shares traditional cardiovascular risk factors, such as hypertension, smoking, diabetes, obesity, obstructive sleep apnea, myocardial infarction, and heart failure. It is associated with significant morbidity and mortality (3). There are robust evidence linking obesity and a high risk of AF. Excess body weight is associated with the development of several cardiovascular conditions that provide the substrate for atrial remodeling and contribute to the initiation and perpetuation of AF (4). Visceral adipose tissue is believed to play a more central role in the pathogenesis of cardiovascular diseases, including AF, than subcutaneous adipose tissue (5). Visceral fat represents a metabolically active organ that, accumulating, can negatively influence the anatomical, and electrical remodeling of the heart through an increased and sustained inflammatory stimulus and oxidative stress. Moreover, visceral fat, also through neurohormonal activation, modulates atrium enlargement and electrical instability, thus compromising the atrial electrophysiology (6, 7).

**FIGURE 1:**
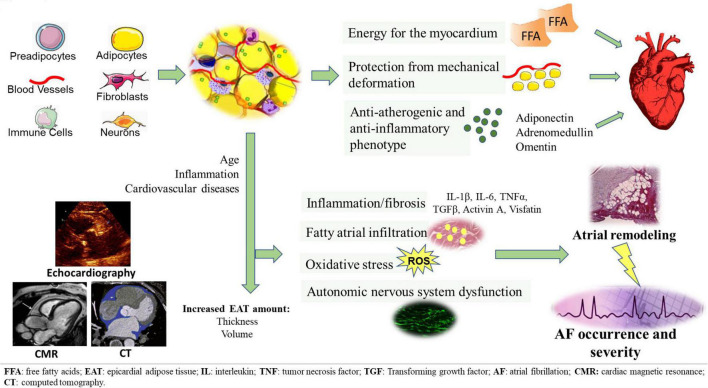
EAT tissue structure and its pathological switch from a protective anti-inflammatory and anti-atherogenic phenotype to a pro-inflammatory, pro-fibrotic, and pro-adrenergic one. EAT potentially contributes to the development of AF through several arrhythmogenic mechanisms, including fatty atrial infiltration, secretion of pro-inflammatory and pro-fibrotic mediators, oxidative stress, autonomic nervous system dysfunction. EAT measurement can be obtained through different imaging methods, such as echocardiography, CT and CMR.

In recent years, the deposit of cardiac visceral fat, known as epicardial adipose tissue (EAT), has aroused considerable scientific interest, due to its peculiar characteristics and its proximity to the heart. Numerous authors have described its role in the pathogenesis of several cardiovascular diseases. A growing body of published data documents the implication of EAT in AF onset and severity. EAT has been proposed as an important factor involved in structural and electrical atrial remodeling, as well as in AF burden (8). Being in contiguity with the underlying myocardium, EAT can directly infiltrate it. EAT is also able to actively secrete numerous pro-inflammatory cytokines and adipokines, capable of adversely affecting the myocardium by paracrine and endocrine manner; in addition, EAT contains abundant ganglionated plexi that contribute to arrhythmogenesis through autonomic nervous system (ANS) stimulation (9). Given the recognized role of EAT in the development and progression of AF, its quantification has been proposed, through different imaging methods, as a useful prognostic tool to predict the presence, severity and recurrence of AF (10). Furthermore, EAT is increasingly emerging as a promising potential therapeutic target (11) (Figure 1).

In this review, we summarize the electrophysiological mechanisms underlying the link between EAT and AF, in order to explore the current knowledge on the arrhythmogenicity of EAT and its role in the pathogenesis of AF.

## Role of Inflammation in Atrial Remodeling and Atrial Fibrillation

Chaotic and ectopic electrical activity and uncoordinated atrial contraction, which characterize AF, are associated with increased risk and severity of stroke, heart failure and increased overall mortality (12). AF can occur as the result of numerous different pathophysiological mechanisms, leading to atrial morphological and electrophysiological alterations. Hemodynamic stress is one of the mechanisms promoting AF, and hypertension is considered an independent predictor of AF and contributes to its progression (13). Structural heart disease has been recognized to play an important role in the development of this arrhythmia, in particular valvular diseases, which result in increased left atrial pressure, hemodynamic stress, and left atrial remodeling (14). Vascular disease, especially coronary artery disease, is frequently found in AF patients, and may be associated with AF-related complications (15). Similarly, heart failure and AF often coexist (15), and they appear to promote each other, since heart failure and left ventricle dysfunction favor pressure overload and atrial dilation, and AF worsens the left ventricle function. The clinical prevalence of AF strongly increases with age. It has been described that the senescent heart is characterized by structural and electrical properties that predispose to AF (16). Diabetes mellitus is another established risk factors for AF and stroke: autonomic dysfunction associated with diabetic cardiomyopathy promotes AF occurrence (17).

All these clinical conditions appear to enhance AF susceptibility by many different mechanisms. The structural and functional changes of the atria promoted by aging, neurohumoral activation, hemodynamic stress and chronic atrial stretch, are the result of the multiple signaling pathways activation leading to myocyte hypertrophy, fibroblast proliferation, and extracellular matrix remodeling (18). This “atrial remodeling” determines an electrophysiological substrate characterized by the interruption of the electrical interconnections between muscle bundles, by shortening of the atrial refractoriness and the reentrant wavelength. It has been described that this proarrhythmic substrate can promote ectopic activity from pulmonary veins or other sites and can trigger AF (18). It is known that inflammation plays a central role in the pathogenesis of AF and in the development of atrial cardiomyopathy (19), as demonstrated by the observation that systemic inflammatory and immune diseases, such as rheumatoid arthritis, psoriasis, and metabolic diseases, increase the risk of developing AF (20–22). Abnormal atrial histology, characterized by inflammatory infiltrates, fibrosis and necrosis of adjacent cardiomyocytes, has been uniformly found in multiple atrial biopsy samples of patients with AF (23). Inflammation and the associated immune response are involved in initiating and maintaining AF, which in turn can further sustain inflammation, thus further promoting the proarrhythmic substrate, thereby perpetuating and increasing the severity of the arrhythmia (24). AF patients show increased levels of pro-inflammatory markers compared to subjects in sinus rhythm, and C-reactive protein (CRP) and Interleukin (IL)-6 have been shown to be associated with AF development, recurrence and burden, and failure rate of cardioversion (25, 26). Atrial fibrosis is believed to be one of the most important factors in promoting the substrate for AF. Lymphomononuclear cells, which are the predominant immune cells that infiltrate the atrial myocardium of AF patients, secrete high levels of tumor necrosis factor (TNF)-α, transforming growth factor (TGF)-β, and IL-6. These mediatorscan strongly contribute to atrial fibrosis and electrical remodeling (27). TNF activates myofibroblasts through the TGF-β signaling pathway and increases the secretion of matrix metalloproteinases (MMP)-2 and MMP-9, which mediate atrial remodeling (28). This evidence is confirmed by the efficacy of anti-TNF antibodies in reducing MMP-2 and MMP-9 activity and preventing collagen synthesis and deposition (29). Leukocyte activation and expression of myeloperoxidase in the atrium promote MMP-2 and MMP-9 activity, contributing to atrial fibrosis and slowing atrial conduction (30). Mast cells, key mediators of immune responses, are critically involved in AF pathogenesis, by increasing the platelet-derived growth factor A (PDGF-A) synthesis and by promoting cell proliferation and collagen expression in cardiac fibroblasts (31). PDGF and its receptor are strongly expressed in atrial fibroblasts and increase atrial fibrosis and susceptibility to AF (32). Clinical studies on patients with AF showed that high circulating levels of CRP and IL-6, and low levels of IL-18 are associated with increased atrial size and duration of AF episodes, thus supporting the role of inflammation in atrial remodeling and AF (33, 34).

## Obesity, Visceral Adipose Tissue, and Atrial Fibrillation

Obesity has been clearly linked to the development of AF. By analyzing data from 2.717 participants of the Health, Aging, and Body Composition Study, it has been reported that increases in body max index (BMI), abdominal circumference, and total fat mass are associated with increased AF risk (35). It has been reported an association between obesity, atrial enlargement and ventricular diastolic dysfunction, both known to be risk factors for AF (36, 37). A meta-analysis by Wanahita et al. reported an increased and progressive risk of developing AF for obese individuals compared to non-obese subjects (36). Obesity has been associated with new-onset AF not only in the general population but even in patients after cardiac surgery (38, 39). Obesity is traditionally considered an important cardiovascular risk factor, related to hemodynamic and metabolic imbalances. Overweight and obesity are very common in patients with diabetes, hypertension and obstructive sleep apnea, which can contribute to the development of AF with different mechanisms, including increased sympathetic tone, left ventricular dysfunction, and increased left atrial size and atrial remodeling (40, 41). Animal studies suggested that excessive myocardial lipid deposits may be an important pathogenetic cause of atrial remodeling, similar to what is observed at the ventricular level during dilated cardiomyopathy (42). Obesity leads to immune cells infiltration into the adipose tissue and to a shift in the macrophages polarization from the M2- anti-inflammatory state to the M1 pro-inflammatory state, that contributes to insulin resistance (43). Several studies reported an increased inflammation of adipose tissue and a greater secretion of proinflammatory mediators, such as TNFα and IL-6, in patients with obesity (44, 45). Furthermore, in obese patients, the accumulation of lipids in cardiomyocytes promotes apoptosis and further increases local inflammation (46). Overall, these evidence support the important contribution of inflammation in mediating the pathophysiological mechanisms of AF in patients with obesity.

More and more evidence indicate that the regional distribution of body fat, rather than general obesity, correlates with systemic inflammation, insulin resistance and oxidative stress. Visceral fat is more metabolically active than subcutaneous fat, thus visceral obesity seems to have a greater unfavorable role than subcutaneous obesity, being associated with enhanced systemic inflammation, a more unfavorable metabolic phenotype, and a greater cardiovascular risk (5, 47, 48). Visceral adipose tissue, mainly located in the abdominal cavity, includes intrahepatic and mesenteric, omental, and retroperitoneal fat, and is considered a risk factor for cardiometabolic diseases (49). The deleterious effects of visceral fat are mainly mediated by inflammation. Several studies demonstrated the association between increased visceral adipose tissue and inflammation, through the enhanced production and secretion of numerous adipokines and pro-inflammatory mediators, involved in altered metabolism and cardiovascular disease. As visceral obesity increases, the expression of IL-6 and monocyte chemoattractant protein (MCP)-1 increases proportionally (50–52). Moreover, it has been reported that levels of certain surrogate inflammatory markers, such as white blood cell, high-sensitivity CRP and neutrophil-lymphocyte, are independently associated with visceral fat and not with subcutaneous adipose tissue, thus supporting the importance of the metabolic contribution of this fat depot (53). The accumulation of abdominal visceral fat correlates with high prevalence of insulin resistance and metabolic syndrome (49). In addition, a close relationship between increased visceral adipose tissue and increased risk of cardiovascular outcomes has been demonstrated (54). Interestingly, a great correlation between the amount of visceral adipose tissue and EAT has been reported, as well as the association between EAT and atherosclerotic risk factors (55, 56). The unique characteristics of this adipose tissue and its proximity to the heart, has attracted considerable interest regarding its role in cardiovascular diseases and its potential proarrhythmic effect.

## Epicardial Adipose Tissue: Pathophysiology and Clinical Assessment

Epicardial adipose tissue represents the visceral fat depot of the heart and is located between the myocardium and the visceral layer of pericardium, mainly distributed in atrio-ventricular and interventricular grooves and along the major branches of the coronary arteries. Histological analysis allowed the characterization of this tissue, mainly composed of small adipocytes, but also stromo-vascular and immune cells, and a variable degrees of leukocyte accumulation, with T lymphocytes, macrophages and mast cells (57). The presence of ganglia and intercommunicating nerves within EAT, and a high production of nerve growth factor has also been demonstrated, thus suggesting a potential role of EAT as a scaffold for cardiac autonomic nerves and ganglionated plexi (57). There are no anatomical boundaries dividing EAT and surrounding tissues, thus resulting in a close proximity between EAT and the myocardium, which share the same microcirculation. The contiguity of these two tissues underlies the strong interaction between EAT and myocardium. EAT is a highly active metabolic organ and, in physiologic conditions, exerts numerous protective functions. It protects the heart and coronary arteries from mechanical deformation and facilitates vessel remodeling (58). EAT has important thermogenic function, by providing free fatty acids (FFAs), the main source of energy for the myocardium, readily available for cardiomyocytes in conditions of increased metabolic demand. Furthermore, EAT may sequester circulating FFAs thus providing protection against lipotoxicity (59). Moreover, EAT is a tissue with relevant endocrine and paracrine properties. It is capable of producing and secreting numerous anti-atherogenic and anti-inflammatory cytokines and directly disseminating them into the myocardium and coronary lumen. The adipocytokines released by EAT include adiponectin, adrenomedullin, and omentin, which exert beneficial effects through their antioxidant, anti-inflammatory, and anti-apoptotic properties (60). Interestingly, it has been demonstrated an association between increased epicardial adiponectin and the maintenance of sinus rhythm after cardiac surgery, thus confirming its protective role for the heart, counteracting inflammation and fibrosis, and consequently the arrhythmogenesis (61). These positive and beneficial properties of EAT are mainly expressed in conditions of health and low inflammation and oxidative stress.

However, aging and various pathological conditions, especially inflammatory and cardiovascular diseases, are associated with increased EAT amount and changes in the local microenvironment. In these conditions, EAT takes a pro-inflammatory, pro-fibrotic and pro-atherosclerotic phenotype and produces and secretes mainly pro-inflammatory cytokines and mediators. In this thicker and dysfunctional EAT, the histological analysis showed an increased inflammatory and immune infiltrate, consisting of T and B lymphocytes, dendritic cells, eosinophils and macrophages, and a cellular polarization characterized by a prevalence of proinflammatory macrophages M1 (62, 63). The role of structural and functional changes of EAT in the pathogenesis of various cardiovascular diseases has been explored and confirmed by several studies (54, 56, 57, 64). EAT contributes to the development and progression of cardiovascular diseases, through the synthesis and secretion of pro-inflammatory mediators and neuro-hormones, such as IL-1β, IL- 6, TNFα, MCP-1, resistin and visfatin, able to act on the myocardium and coronary vessels in a paracrine and vasocrine manner, thus negatively influencing the structure and function of the heart. Furthermore, the increase in EAT appears to be associated with a worse cardiovascular outcome (10, 65–67).

The central role of EAT in cardiovascular diseases justifies the considerable interest in the improvement of imaging methods useful for its quantification. In clinical practice both echocardiography, computed tomography (CT) and cardiac magnetic resonance (CMR), are available for EAT quantification and the measurement of EAT has been proposed as a useful marker for cardiovascular and metabolic risk assessment (10). CMR imaging has great diagnostic potential, providing an accurate volumetric measurement of EAT, and so it represents the gold standard for quantifying EAT (68). However, CMR imaging is not readily available in clinical practice, as it is an expensive and time-consuming procedure, so it is difficult to be routinely proposed (69). CT provides an accurate measurement of EAT thickness, volume and total area, with a higher spatial resolution than both echocardiography and CMR imaging, and with high specificity and sensitivity of the measurements, thanks also to the three-dimensional image reconstruction with multidetector-row CT. Antonopoulos et al. developed the perivascular CT fat attenuation index (FAI) in the context of coronary artery disease, providing a characterization, in terms of adipocyte size and lipid content, of the adipose tissue surrounding the coronary arteries. They showed a larger perivascular FAI in unstable plaques than stable plaques and a greatest FAI directly adjacent to the inflamed coronary artery. Thus, the authors proposed the perivascular FAI as a useful, non-invasive method for monitoring vascular inflammation and the development of coronary artery disease (70). The quantitative assessment of coronary inflammation through the perivascular FAI could be a useful tool to improve the prediction of cardiac risk (71). While the FAI index has been tested and validated in the context of coronary heart disease, it certainly offers an attractive possibility of developing a similar index for AF and correlating it with AF patients outcome. The main limitations and disadvantages of CT are related to the high costs of the method, which requires experienced and qualified operators and advanced machines, and the use of ionizing radiation, which limits the use of CT in routine clinical practice (69, 72). Echocardiography allows to overcome these limitations, offering a safe, economical and easily repeatable method for assessing the EAT thickness, and does not expose the patient to the risk of ionizing radiations (73). Among the various proposed echocardiographic methods of quantifying EAT, its measurement at the level of the Rindfleisch fold, between the free wall of the right ventricle and the anterior surface of the ascending aorta, was recently validated against CMR (74). Overall, EAT quantification has been proposed along with traditional predictors, as a new imaging marker for cardiovascular risk stratification and cardiovascular outcome prediction (75–77).

## Epicardial Adipose Tissue and Atrial Fibrillation

It was postulated that EAT can alter ion currents and electrophysiological properties, causing myocardial electrical substrate for AF (78). Following these hypotheses, several authors investigated the potential role of EAT in AF, assessing EAT accumulation by using the different available non-invasive imaging methods, and evaluating the associations between EAT and AF occurrence, severity and outcome (Table 1).

**TABLE 1:** Associations between EAT and AF occurrence, severity and outcome.

References	Study population	EAT measure	Main finding
Maeda et al. (89)	218 AF patients undergoing AF ablation; Mean age ± SD: 64 ± 10.1; Male 74.8%;	EAT Volume at multidetector CT	EAT volume is a predictor of post-ablation recurrence of AF HR: 1.02 (95% CI: 1.00–1.03) *P* = 0.012
Tsao et al. (90)	115 subjects; Mean age ± SD: 63.5 ± 8.7; Male 75%; Sinus rhythm: 20 (17%); AF patients with stroke: 27 (24%); AF patients without stroke: 68 (59%)	EAT Volume at 64-slice multidetector CT	EAT volume is independently associated with the risk of AF-related stroke; OR: 1.12 (95% CI: 1.06–1.19) *P* < 0.001 EAT volume correlates to contractile dysfunction of the left atrium (*r* = –0.369, *P* < 0.001) and circulatory stasis of the atrial appendix (*r* = –0.466, *P* < 0.001).
Chu et al. (91)	190 persistent AF patients Mean age ± SD: 70 ± 10; Male 67%;	EAT Thickness at echocardiography	EAT thickness is associated with worse cardiovascular outcome: cardiovascular mortality, hospitalization for heart failure, myocardial infarction, and stroke; OR 1.224, 95% CI: 1.096–1.368, *P* < 0.001
Muhib et al. (82)	62 patients with hypertrophic cardiomyopathy: Mean age ± SD: 56.8 ± 14; Male 58%; Sinus rhythm: 52 (84%); AF patients: 10 (16%)	EAT Area at CMR	Increased EAT area is significantly related to the presence of AF, independently of sex, age and BMI OR: 1.28 (95% CI: 1.01–1.63) *P* = 0.04
Tsao et al. (80)	102 subjects; Mean age ± SD: 54.4 ± 8.7; Male 71,5%; Sinus rhythm: 34 (33%); AF patients: 68 (67%)	EAT Volume at 64-slice multidetector CT	EAT amount is associated with AF occurrence: EAT volume is significantly increased in patients with AF compared to controls (*P* < 0.01); Increased EAT is independently related to AF recurrence after ablation (*P* = 0.038).
Wong et al. (88)	130 subjects; Mean age ± SD: 56 ± 7,5; Male 71,8%; Sinus rhythm: 20 (15%); AF patients undergoing first-time AF ablation: 110 (85%)	EAT Volume at CMR	EAT volume is associated with the presence [OR: 13.28 (95% CI: 2.23–79.98) *P* = 0.005] and the severity of AF [OR: 3.28 (95% CI: 1.25–8.59) *P* = 0.015], left atrial volumes (*r* = 0.49 *P* < 0.01) and poorer outcomes after AF ablation (*p* = 0.035 by log-rank test).
Shin et al. (83)	160 subjects; Mean age ± SD 51.6 ± 12; Male 72,5%; Sinus rhythm: 80 (50%); Paroxysmal AF: 40 (25%); Persistent AF: 40 (25%)	EAT Volume and Thickness at multislice CT	EAT Volume and periatrial EAT Thickness are significantly larger in AF patients compared to controls and are closely related to the chronicity of AF (*P* < 0.01)
Thanassoulis et al. (79)	3217 individuals from the Framingham Heart Study Mean age ± SD 50.6 ± 10.1; Male 52%; AF: 54 (1,7%)	EAT Volume at multidetector CT	EAT volume is associated with the prevalence of AF, independently by traditional AF risk factors, including BMI OR: 1.28 (95% CI: 1.01–1.63) *P* = 0.04
Al Chekakie et al. (81)	273 subjects; Mean age ± SD: 57 ± 12.3; Male 67%; Sinus rhythm: 76 (27,8%); Paroxysmal AF: 126 (46,1%); Persistent AF: 71 (26%)	EAT Volume at 64-slice multidetector CT	EAT volume is associated with AF, independently of traditional risk factors including BMI and left atrial enlargement OR: 1.13 (95% CI: 1.03–1.24) *P* = 0.01 EAT volume is larger in patients with persistent AF compared to patients with paroxysmal AF or sinus rhythm (*P* < 0.01)
Batal et al. (8)	169 subjects; Mean age ± SD: 54.6 ± 13.2; Male 65,1%; Sinus rhythm: 73 (43,2%); Paroxysmal AF: 60 (35,5%); Persistent AF: 36 (21,3%)	EAT Thickness at 64-slice multidetector CT	Increased left atrium EAT thickness is associated with AF burden independently of age, BMI or left atrium area OR: 5.30 (95% CI: 1.39–20.24) *P* = 0.015

*EAT, epicardial adipose tissue; CT, computed tomography; AF, atrial fibrillation; BMI, body max index; CMR, cardiac magnetic resonance; SD, standard deviation; 95% CI, 95% confidence interval; OR, odds ratio; HR, hazard ratio; r, correlation (Pearson or Spearman).*

A CT analysis of 3217 individuals from the Framingham Heart Study showed the correlation between EAT volume and the prevalence of AF. Interestingly, this association resulted to be independent by traditional AF risk factors, including BMI (79). In addition to the presence of AF, EAT amount also correlates with its severity and progression. Tsao and colleagues (80) quantified the EAT accumulation by CT and demonstrated a significantly larger volume of EAT surrounding the left atrium in patients with AF and also reported a correlation between EAT amount and AF severity: patients with persistent AF showed larger EAT volumes than those with paroxysmal AF. In contrast, there was no association between BMI and AF severity. The same conclusions were reached by Al Chekakie et al. (81) who demonstrated a higher EAT volume in patients with persistent AF compared to patients with paroxysmal AF or sinus rhythm and by Batal et al. (8), who suggested that EAT can promote AF persistence. Muhib et al. (82) used CMR to assess EAT accumulation in patients with hypertrophic cardiomyopathy and showed similar results, reporting an association between the increased EAT area and the incidence of AF independent of clinical risk factor, such as sex, age, and BMI. Some researchers also investigated the correlation between the regional distribution of EAT and the development of AF, by measuring the EAT total volume, and the thickness of periatrial and periventricular EAT by multislice CT. They found that EAT total volume and periatrial EAT thickness, and not the periventricular EAT thickness, were significantly larger in AF patients compared to controls and were closely related to the chronicity of AF (83). The strength of the association between EAT amount and atrial arrhythmias has been further expanded by the results of two meta-analysis (84, 85). Wong et al. (84) confirmed the relationship between EAT and AF, suggesting that it is stronger than the association between AF and abdominal or overall adiposity. Gaeta et al. (85), based on the analysis of seven imaging studies, compared patients with persistent AF, patients with paroxysmal AF and healthy subjects. They reported a higher EAT volume in patients with persistent and paroxysmal AF than in healthy subjects. Moreover, patients with persistent AF showed a significant increase in EAT volume compared to patients with paroxysmal AF, thus further supporting the association between EAT amount and AF severity.

Epicardial adipose tissue is thought also to influence cardiovascular outcome (86, 87), and the correlation between EAT and cardiovascular outcome has been investigated also in AF population. A strong relationship between EAT and AF recurrence after catheter ablation has been described (80, 88, 89). Increased EAT volume is associated with worse outcomes and early AF recurrence after ablation, thus leading to a lower catheter ablation efficacy. Interestingly, traditional systemic measures of adiposity, such as BMI and body surface area, appear to be not associated with these outcomes. These findings are consistent with the hypothesis of a local pathogenic and pro-arrhythmic effect of EAT (80, 88). Similarly, Maeda et al. (89), evaluated the impact of EAT volume, assessed by using multidetector CT, on recurrent AF after radiofrequency ablation. These authors suggested EAT volume as new predictor of AF recurrence after catheter ablation. The correlation between EAT accumulation and cardiovascular outcome in AF patients has been investigated also by Tsao et al. (90), who used 64-slice multidetector CT to assess the periatrial EAT volume, and reported an independent association between increased EAT volume and stroke, thus suggesting the EAT assessment as a useful tool for grading the risk of cardioembolic stroke in AF patients. Interestingly, a relationship between the amount of EAT and contractile dysfunction of the left atrium and circulatory stasis of the atrial appendix, two recognized risk factors for AF-related stroke, has been described. Similarly, the negative prognostic role of echocardiographic EAT thickness in AF patients has been also reported. EAT thickness resulted to be related to cardiovascular hospitalization and mortality, myocardial infarction and stroke. Interestingly, EAT thickness appears to have an additive predictive power for cardiovascular risk when added to traditional risk predictors, such as CHA2DS2-VASc score, left atrial volume, and systolic and diastolic left ventricle function (91).

The relationship between EAT and onset, severity and recurrence of AF is the result of the complex crosstalk between EAT and the neighboring atrial myocardium (92). EAT potentially contributes to the development of AF through several mechanisms, including fatty atrial infiltration, inflammation, production and release of reactive oxygen species (ROS) and ANS dysfunction. These mechanisms are probably further exacerbated during cardiac surgery and recent evidence suggest that EAT inflammatory status could be related to post-operative AF occurrence (93–95).

The ability of EAT to penetrate the myocardium and generate atrial fatty infiltrates has been widely demonstrated by histological examinations of atrial samples obtained from both humans and sheep model of persistent AF (96). A correlation between the characteristics of atrial myocardium fatty infiltrates and the history of AF has also been described. Fatty infiltration into the atria with persistent AF appears to be greater than atria with paroxysmal AF (97). Moreover, the fibrosis of atrial fatty infiltrates seems to be associated with the presence and severity of AF: fibro-fatty infiltrates predominate in patients with permanent AF compared with paroxysmal AF, emphasizing its importance in arrhythmogenicity (97). This structural atrial remodeling seems to be attributed to the activity of cytotoxic T lymphocytes, observed in human atria together with adipocyte cell death, and constitutes a substrate for AF (96). Fatty infiltration into the myocardium contributes to the arrhythmogenesis by promoting cardiac conduction abnormalities and the creation of re-entry circuits (8). Inflammation has been proposed as one of the main pathogenetic mechanisms linking EAT and AF. In pathologic conditions EAT becomes a relevant source of inflammatory cytokines, such as TNFα, IL-1β, IL-6 and IL-8, that may influence the adjacent atrial myocardium and facilitate arrhythmogenesis (98, 99). Several studies have reported the association between the increase in inflammatory markers, such as CRP, TNF-α, IL-6, IL-8, IL-1β, and AF presence and severity (24–26, 100) and cytokines secreted by EAT may actively contribute to this pro-inflammatory status observed in AF patients (101). The 18-fluorodeoxyglucose (FDG)-positron emission tomography (PET)/TC has been used by Mazurek et al. to examine the inflammatory activity of EAT and to compare it in patients with and without AF. EAT tracer uptake was higher in AF patients than in controls, thereby reflecting an increased EAT inflammatory activity in AF patients. Interestingly, subcutaneous adipose tissue and other visceral adipose tissue depots showed a lower uptake with respect to EAT, thus supporting the role of EAT as the main active adipose tissue potentially involved in the inflammatory substrate of AF (101, 102). The induction of oxidative stress is another proposed mechanism to explain the involvement of EAT in AF pathogenesis. EAT is richer in ROS than other fat depots, thus it is considered the main source of ROS, with detrimental local effects on adjacent atrial myocardium (103). It is widely recognized that oxidative stress plays a central role in AF pathogenesis (104, 105), as suggested by the observation that ROS inhibition by antioxidants in animal models attenuates atrial remodeling (106). Furthermore, EAT is a relevant source of pro-fibrotic factors, such as TGF-β1, activin A, a member of the TGF-β superfamily and MMP2, MMP7, key regulators of extra-cellular matrix activity. These mediators resulted to be up-regulated in AF and contribute to atrial collagen deposition, fibrosis and remodeling (107–109), thus supporting the role of EAT in promoting atrial remodeling and altering atrial electrophysiological properties. Further involvement of EAT in the promotion of AF appears to be linked to the production of aromatase, an enzymatic protein that converts androgens into estrogen, and which appears to play an important role in susceptibility to atrial arrhythmias through modulation of electromechanical properties. The increase in EAT amount is associated with greater local aromatase effect, resulting in enhanced aromatase estrogenic capacity and atrial arrhythmogenicity. The occurrence/duration of triggered atrial arrhythmias results to be significantly enhanced with the increase of the total aromatase content of EAT, thus suggesting the role of this enzymatic EAT-derived protein in promoting AF (110). In addition to these pathogenetic mechanisms, EAT could sustain the susceptibility to atrial arrhythmias by promoting ANS dysfunction. The dysregulated production and secretion of adipokines by adipose tissue, particularly by visceral fat depots, may stimulate central sympathetic nervous system (SNS) activity (111) and several studies reported a close relationship between EAT and myocardial autonomic function (112, 113). Experimental studies demonstrated that EAT is a site of catecholamines biosynthesis and an important source of norepinephrine. Through the secretion of catecholamines, EAT may directly contribute to an increased sympathetic tone and to a sympatho-vagal imbalance, thereby promoting atrial arrhythmias. Interestingly, catecholamine levels, as well as expression of catecholamine biosynthetic enzymes, resulted to be higher in EAT than in subcutaneous adipose tissue. These data support the adrenergic activity of EAT and its potential role in AF (114). Moreover, EAT contains abundant both adrenergic and cholinergic nerves that interact with the extrinsic nervous system and para-SNS. In response to extrinsic nerve activation, it occurs a simultaneous activation of these nerve structures within EAT, that results in enhanced triggered activity and facilitates the development of cardiac arrhythmias (111). In addition, it has been hypothesized that an increased EAT amount may alter the function of ganglionated plexi, located near the pulmonary veins, thus promoting AF by induction of spontaneous, rapid and repetitive electrical activity (115). A significant amounts of ganglionated plexi have been identified also within EAT, and their activation seems to contribute to arrhythmogenesis through ANS stimulation. The efficacy of botulinum toxin injection into EAT in suppressing atrial tachyarrhythmia seems to be potentially related to the inhibition of ganglionated plexi, thus supporting the hypothesis of their involvement in AF occurrence (116). Interestingly, it has been reported a correlation between EAT thickness and ANS dysfunction (112), and a reduction of ANS activity after catheter ablation of EAT, thus offering an interesting therapeutic perspective (117). Table 2 shows the main inflammatory mediators and adipokines secreted by EAT and their contribution to arrhythmogenesis.

**TABLE 2:** The main EAT-secreted mediators and their contribution to arrhythmogenesis.

Mediators	Action
IL-1β, TNF-α, IL-6, IL-8	Local pro-inflammatory status Atrial remodeling: collagen deposition and fibrosis Ion Channel Modulation
Activin A, MCP-1, TGF-β MMP2, MMP9, PDGF	Atrial remodeling: collagen deposition and fibrosis Altered atrial conduction
ROS	Ion Channel Modulation Vascular and cardiac endothelial cells dysfunction
Aromatase	Increased local cardiac estrogen synthesis Influence on electromechanical properties and myocyte viability signaling
Catecholamines	Increased sympathetic tone Sympatho-vagal imbalance

*EAT, epicardial adipose tissue; IL, interleukin; TNF, tumor necrosis factor; MCP, monocyte chemoattractant protein; TGF, transforming growth factor; MMP, matrix metalloproteinases; PDGF, platelet-derived growth factor; ROS, reactive oxygen species.*

## Eat as a New Potential Therapeutic Target

Given the important role of EAT in the development and progression of cardiovascular diseases, it has been proposed as a promising therapeutic target. A recent systematic review and meta-analysis provided evidence that lifestyle changes consisting in exercise and diet, as well as bariatric surgery and pharmaceutical interventions can reduce EAT volume (10).

Several authors investigated the efficacy of lifestyle interventions, including exercise and dietary restrictions, in reducing EAT amount. Konwerski et al. (118) reported a lower EAT volume, assessed at CMR, in ultramarathon runners compared to a control group of sedentary subjects. Furthermore, the ultramarathon runners showed to have lower circulating levels of IL-6 and a more favorable lipid profile compared to the control group (118). Christensen et al. evaluated the effects of physical activity on EAT amount, assessed by CMR, in physically inactive people with abdominal obesity. They showed that both endurance and resistance training can reduce EAT mass up to 32% compared with the no-exercise control group (119). Two recent systematic reviews and meta-analyses further strengthen the effectiveness of physical activity in reducing EAT volume, thus confirming the role of exercise interventions in favorably modulating EAT volume and metabolic profile (120, 121). Also, dietary restrictions can significantly reduce EAT volume. A weight loss interventional study has been conducted to evaluate the weight loss-induced changes in EAT and in other regional fat compartments in obese men. In this study, the low-calorie diet-induced weight loss was associated to the reduction of EAT thickness, assessed by echocardiography, thus suggesting that dietary restriction may represent an effective non-pharmacological strategy for targeting EAT (122). These results are in line with previous evidence showing a significant decrease in echocardiographic EAT thickness in obese subjects, up to 33%, after 6-month of very low-calorie diet, confirming that EAT may be particularly responsive to dietary restrictions (123).

Bariatric surgery has been proposed as another strategy to reduce EAT volume. Willens et al. examined the effects of weight loss after bariatric surgery on EAT amount in patients with severe obesity and showed that EAT thickness, assessed by echocardiography, decreases in patients who have substantial weight loss after bariatric surgery, thus suggesting the echocardiographic EAT thickness measurement to monitor visceral fat loss during weight reduction therapies (124). Interestingly, in obese subjects, it has been demonstrated a reduction in AF burden post bariatric surgery, compared with usual care (125–127). The beneficial effect of bariatric surgery on development of AF seems to be linked to the attenuation of various hemodynamic, metabolic and inflammatory stimuli, able to favorably influence the structure and function of the heart so as to reduce the risk of arrhythmia (128). It is unclear whether the reduction in the risk of AF can be at least partly explained by the reduction and modulation of EAT. Further studies are needed to confirm this hypothesis.

In addition to these weight-loss therapeutic strategies, several studies indicate that EAT volume may be affected by pharmaceutical interventions, including antidiabetic therapies and statins. The effects of antidiabetic drugs on EAT have been thoroughly investigated. A prospective study analyzed the potential positive effect of metformin on EAT thickness, measured by echocardiography, in 40 newly diagnosed type 2 diabetes patients and reported a statistically significant decrease in EAT thickness after 3 months of metformin monotherapy (129). The anti-inflammatory, thermogenic and metabolic properties of metformin are likely to underlie its interaction with EAT (130). Interestingly, the ability of metformin to reduce EAT secretion of the proinflammatory cytokine, activin A, has been described (131). Similarly, thiazolidinediones were shown to reduce EAT inflammation through reduction of mast cell and macrophage infiltrates and by improving vascularity (132). Thiazolidinediones can therefore favorably affect the secretome of EAT by reducing the production of proinflammatory cytokines (133). However, thiazolidinediones are associated with weight gain and risk of heart failure, which often complicates the clinic of AF patients. These side effects require considerable care and limit the use of these drugs in this type of patients (134). Among antidiabetic drugs, also the effects on EAT of sodium-glucose cotransporter 2 (SGLT2) inhibitors, glucagon-like peptide-1 (GLP-1) agonists, and dipeptidyl peptidase-4 (DPP-4) inhibitors have been investigated primarily for their anti-inflammatory properties on adipose tissue. Sato et al. (135) evaluated the effect of SGLT2 inhibitors on EAT volume, assessed using CT, and on atrial rhythm disturbance susceptibility, assessed by changes in P-wave indices at electrocardiogram. They showed that dapagliflozin treatment reduced EAT volume and P-wave indices, in patients with type 2 diabetes mellitus and coronary artery disease. Interestingly, the change in EAT volume was an independent determinant of the change in P-wave dispersion (135). Another study reported the positive effect of dapagliflozin, able to cause a rapid and significant EAT thickness reduction, that was independent of weight loss (136). These results are reinforced by a systematic review and meta-analysis reporting that the amount of EAT is significantly reduced in type 2 diabetes mellitus patients with SGLT2 inhibitors treatment (137). The exact mechanism underlying the effectiveness of SGLT2 inhibitors on EAT is yet to be clarified. However, in patients with cardiovascular disease, it has been demonstrated that dapagliflozin improves EAT cells’ sensitivity to insulin, thus increasing glucose uptake, reduces the secretion of pro-inflammatory chemokines, and improves the differentiation of EAT cells (138). The reduction of EAT amount has been reported also for GLP-1 agonists and DPP-4 inhibitors treatment (139, 140), probably through their anti-inflammatory and antioxidant properties (141–143).

Great interest has been paid to the potential role of statins in positively modulating the amount and inflammatory profile of EAT. A sub-analysis of a randomized trial involving 420 postmenopausal hyperlipidemic women treated with atorvastatin 80 mg/day or pravastatin 40 mg/day showed the efficacy of statin therapy in reducing EAT volume and the greater efficacy of intensive care than moderate-intensity therapy (144). This effect did not seem linked to low-density lipoprotein lowering; thus, the authors suggest that other actions of statins such as anti-inflammatory properties, can justify these results. The association between statin therapy and EAT reduction has been reported also in patients with aortic stenosis (145). Of note, EAT thickness decrease was paralleled by an attenuation of EAT inflammatory profile. Moreover, an *in vitro* experiment has been conducted to evaluate the effects of atorvastatin on EAT and subcutaneous adipose tissue biopsies. Interestingly, atorvastatin showed a direct anti-inflammatory effect on EAT which was significantly higher compared to the subcutaneous adipose tissue response to statin incubation. This evidence suggested EAT as a potential new therapeutic target for statin therapy, able to modulate both EAT thickness and its inflammatory status (145). Raggi et al. showed that statin therapy induces a decrease in EAT attenuation on CT, considered a marker of inflammation, regardless the intensity of low-density lipoprotein cholesterol lowering and with a neutral effect on subcutaneous adipose tissue. These observations suggest that the modulation of EAT by statins may be mediated by their pleiotropic effects through the reduction of cellularity, vascularity and inflammation (146). The efficacy of statin therapy in reducing EAT volume has been described even in patients with AF (147). Some evidence has shown that treatment with atorvastatin counteracts atrial remodeling in a rabbit model of AF (148) and is associated with improved left atrial function in patients with paroxysmal AF (149), thus suggesting potential antiarrhythmic effect of statins. The potential of atorvastatin administration to improve cardiac fibrosis, as observed in a model of hypertensive diastolic heart failure, can strongly underlie its antiarrhythmic properties (150). The antiarrhythmic effect of statins, associated with reduced incidence and recurrence of AF, has been further confirmed by two different meta-analyses (151, 152). Further studies are needed to clarify the potential contribution of EAT modulation in determining this favorable statin effect. Recently, a significant EAT thickness reduction has been reported after 6 months of treatment with the proprotein convertase subtilisin/kexin type 9 inhibitors (PCSK9), a novel group of effective drugs for lowering LDL cholesterol and cardiovascular events (153). Overall, although the underlying mechanisms of action are not yet well clarified, all this evidence may suggest that lipid-lowering therapies, such as statins and PCSK-9 inhibitors, may exert their pleiotropic and antiarrhythmic effects at least in part through EAT amount and metabolic activity modulation.

In summary, the effectiveness of lifestyle changes, bariatric surgery and pharmaceutical interventions in reducing EAT amount has been widely recognized. However, to date, there is insufficient evidence regarding the positive impact of the EAT reduction following these therapeutic strategies on the patient’s clinical outcome. The aim of future studies should be to clarify this aspect.

## Future Directions

In this review we described the recent evidence exploring the potential role of EAT, the visceral fat depot of the heart, in the pathogenesis of AF, by analyzing the mechanisms by which EAT can promote structural and electrical atrial remodeling and the potential therapeutic strategies targeting the cardiac visceral fat. The association between EAT and AF is now strongly recognized and well established; however, the exact mechanisms underlying this association are not fully defined. Therefore, further studies are required in this regard. The elucidation of the mechanisms by which EAT can alter local electrophysiology is essential to improve risk assessment and prevention of cardiac arrhythmias, and for the development of specific therapeutic strategies. Although several researchers have reported encouraging results of different EAT-targeted therapies, more studies are needed to allow its introduction into clinical practice and to shed light on the potential impact of the EAT-targeted therapies on patient clinical outcome. Overall, future studies should elucidate mechanisms by which EAT is involved in atrial cardiomyopathy, identify new potential therapeutic target and test clinical benefits of EAT reduction.

## Conclusion

Atrial fibrillation is a progressive and multifactorial arrhythmia, that occurs as the result of numerous different pathophysiological mechanisms, leading to atrial morphological and electrophysiological alterations. Among the recognized risk factors of AF, obesity and visceral fat accumulation play an important role in promoting the proarrhythmic substrate, thereby predisposing to development of AF. Numerous evidences indicate the close association between EAT, the visceral fat depot of the heart, and AF. EAT can exerts its proarrhythmic properties by several mechanisms, such as inflammation, oxidative stress, fatty infiltration, fibrosis and ANS stimulation, all factors implicated in structural and electrical atrial remodeling. Therefore, EAT, by promoting the formation of AF substrate, is thought to predispose to AF and to enhance the severity and the perpetuating of the arrhythmia. Several imaging methods have been introduced into clinical practice and allow an accurate measurement of the EAT thickness and volume. EAT quantification has been proposed, in addition to traditional predictors, as a new imaging marker useful for both risk stratification and prediction of cardiovascular outcome. Furthermore, given its crucial role in the development and progression of cardiovascular diseases, several authors proposed EAT as a new promising therapeutic target. Although the encouraging results of several therapies targeting EAT, to date, there are no approved specific therapies for EAT in clinical practice. Elucidating the pathophysiologic mechanisms by which EAT can alter local electrophysiology and promote AF substrate will provide insight for improved risk assessment and prevention of AF, and for development of specific therapeutic strategies.

## Author Contributions

MC and VP conceived the manuscript structure and wrote the manuscript with support from PP. LP and VV contributed to write and revised the section “Role of inflammation in Atrial Remodeling and Atrial Fibrillation.” SC and AF contributed to write and revised the sections “Obesity, Visceral Adipose Tissue, and Atrial Fibrillation” and “EAT as a New Potential Therapeutic Target.” EA and VR contributed to write and revised the section “Epicardial Adipose Tissue: Pathophysiology and Clinical Assessment.” EP and PF contributed to write and revised the section “Epicardial Adipose Tissue and Atrial Fibrillation.” VP and DL supervised other authors and contributed to the final version of the manuscript. All authors contributed to the article and approved the submitted version.

## Conflict of Interest

The authors declare that the research was conducted in the absence of any commercial or financial relationships that could be construed as a potential conflict of interest.

## Publisher’s Note

All claims expressed in this article are solely those of the authors and do not necessarily represent those of their affiliated organizations, or those of the publisher, the editors and the reviewers. Any product that may be evaluated in this article, or claim that may be made by its manufacturer, is not guaranteed or endorsed by the publisher.
